# 

**Published:** 1883-02

**Authors:** 


### CONTENTS.

| | Page. | | Page. |
|---|---|---|---|
| Pneumonia | 33 | Hereditary Disease | 49 |
| Care of Children | 35 | Practical Knowledge | 50 |
| Health Capacity | 35 | Small Pox | 52 |
| Facts Worth Remembering | 36 | Spring Diseases | 54 |
| A Lay Sermon | 38 | To Cure a Cold | 55 |
| Scientific Notes | 40 | Dieting for Health | 56 |
| Sea Weed as an Anti-Fat | 40 | Bad Temper and Insanity | 57 |
| Feeding Horses | 41 | Hardening the Constitution | 58 |
| Pure Water | 43 | Finger Nails | 58 |
| How to Get Pure Water | 44 | Cold Feet | 59 |
| Human Health | 46 | A Natural Wish | 61 |
| The Teeth | 47 | Health | 61 |
| The Nature of Consumption | 49 | Croup | 62 |

